# Late Holocene droughts and cave ice harvesting by Ancestral Puebloans

**DOI:** 10.1038/s41598-020-76988-1

**Published:** 2020-11-18

**Authors:** Bogdan P. Onac, Steven M. Baumann, Dylan S. Parmenter, Eric Weaver, Tiberiu B. Sava

**Affiliations:** 1 School of Geosciences, University of South Florida, Tampa, FL 33620 USA; 2 Emil G. Racoviță Institute, Babeș-Bolyai University, 400006 Cluj-Napoca, Romania; 3 National Park Service, El Malpais and El Morro National Monuments, Grants, NM 87020 USA; 4 Department of Earth Sciences, University of Minnesota, Minneapolis, MN 55455 USA; 5 Horia Hulubei National Institute for Physics and Nuclear Engineering, 077125 Măgurele, Romania

**Keywords:** Palaeoclimate, Archaeology

## Abstract

Water availability for Native Americans in the southwestern United States during periods of prolonged droughts is poorly understood as regional hydroclimate records are scant or contradicting. Here, we show that radiocarbon-dated charcoal recovered from an ice deposit accumulated in Cave 29, western New Mexico, provide unambiguous evidence for five drought events that impacted the Ancestral Puebloan society between ~ AD 150 and 950. The presence of abundant charred material in this cave indicates that they periodically obtained drinking water by using fire to melt cave ice, and sheds light on one of many human–environment interactions in the Southwest in a context when climate change forced growing Ancestral Puebloan populations to exploit water resources in unexpected locations. The melting of cave ice under current climate conditions is both uncovering and threatening a fragile source of paleoenvironmental and archaeological evidence of human adaptations to a seemingly marginal environment.

## Introduction

**Figure 1:**
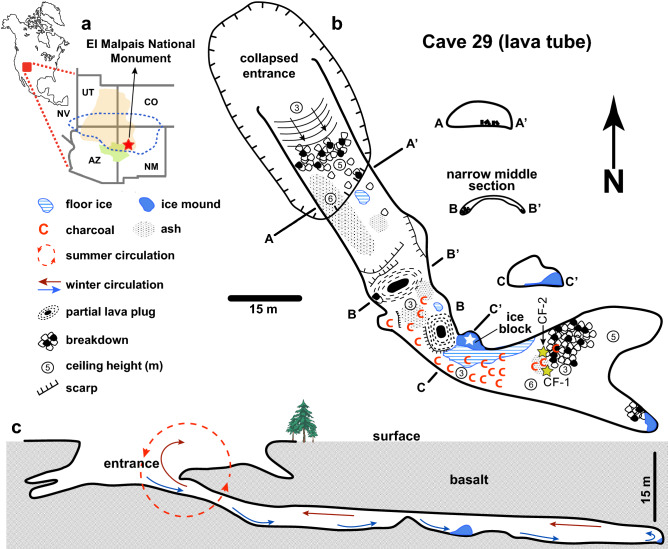
Map of Cave 29. (**a**) Site location in western New Mexico, USA (red star). The approximate boundaries of the Colorado Plateau (brown shading), Zuni land (green shading) and Ancestral Pueblo culture area (blue dashed line)^1^ are also shown. The maps were created by the Generic Mapping Tool (GMT v. 6.1 from https://www.generic-mapping-tools.org^22^. (**b**) Plan of the cave showing the location of ice and charcoal deposits. White star denotes the coring site. (**c**) Schematic profile displaying the pattern of summer and winter air circulation within the cave. Map (**b**) and cross-section (**c**) courtesy of V.J. Polyak & R. Knapp.

The southwest United States (hereafter the Southwest) has some of the best known and most conspicuous archeological sites in North America (Refs.^1,2^ and Supplementary Information S1). One of the controlling factors in the development of the cultural mosaic in the pre-contact Southwest was water availability^3,4^. The type of water resource, as well as its accessibility and reliability, strongly influenced settlement and subsistence strategies, agricultural intensification, demographic trends, and migration from the end of the Late Archaic through the early Pueblo periods (AD 100–900) in the Zuni-Acoma area of the Little Colorado River drainage^5^. The type of water resource and often its context also can have ritual significance to bring rain, for the preparation of medicine and for ceremony. Water from certain contexts such as springs and caves engendered ritual responses involving emblematic connections through pilgrimages and ceremonial activities to obtain the water^6–8^. Archeological evidence suggests periodic altitudinal dislocations trending toward increased population growth and dispersal through upland environments^9–11^. The trend increases gradually from the pre-ceramic (early Formative Basketmaker) through early Pueblo periods, and even more dramatically after AD 850–900^11^. Temporal correlations between population movements in the Zuni-Acoma area from AD 100–950 and shifts in climate have been proposed, but the range and nature of short- and long-term environmental variability that potentially had an impact on the residential mobility of early Puebloans is poorly understood due to limited paleoenvironmental records^12–14^. Of the climate proxy records used to assess past precipitation and temperature in the southern Colorado Plateau, tree rings are among the most important^15,16^. They provide some of the most detailed studies on severe droughts and link Puebloan site occupation with periods of higher moisture^13,14^. Here, we contribute to this line of research by documenting five drought events over an 800-year period using well-dated charcoal fragments preserved in an ice core recovered from a lava tube in the El Malpais National Monument (hereafter ELMA; western New Mexico, Fig. 1a). Corroborating the stratigraphic and paleoclimatic context of the charred material, we provide unambiguous evidence that Ancestral Puebloans used melted ice for drinking water as early as 2000 years ago. Although the use of fire by pre-contact populations to melt ice in the lava tubes was earlier suggested in ELMA^17–19^, to our knowledge, this is the earliest directly dated proof for in-cave burning practices to secure water in the Southwest. While this study focuses on a single lava tube, it illustrates a general methodological approach that links water resource availability with aspects of social variability caused by repetitive depopulation-repopulation of some settlement locations due to migrations across the landscape.

## Results

### Lava tubes and perennial ice deposits

ELMA is located on the southeast edge of the Colorado Plateau (Fig. 1a) and is dominated by basalt flows originating from the Zuni-Bandera volcanic field (Supplementary Fig. S1). Details on the geology, present-day climate, and forest fire history are available in the Supplementary Information S2. Of the 453-lava tubes so far documented for ELMA, 94 have seasonal and/or perennial ice or have had ice at one time, as indicated by bathtub ring deposits of calcite along the lava tube walls and breakdown piles (see Supplementary Information S3). While archeological material has been found in many of ELMA’s lava tubes, virtually all having ice formations contain archeological deposits. The evidence, found in both the twilight zone near entrances as well as deep in the tubes, includes charcoal, charred wood and ash, torches, Puebloan ceramics, lithic artifacts, animal bone, and other material culture along with physical modification of natural surfaces to create drip wells and architectural features. Among these, the only one in which the ice deposit received a thorough investigation, but not to document ice melting activities, is Candelaria Ice Cave, a touristic attraction situated a few km east of our site^20^.

Cave 29 opens at an elevation of 2268 m above sea level, in a section where the ceiling of a more complex lava tube collapsed. The accessible segment has a total surveyed length of 171 m and a depth of ~ 14 m. Overall, the gallery is wide and high (15 × 9 m), except for its middle passage where a massive rafted boulder and breakdown fills almost the entire volume creating a short (5 m) narrow constriction (B-B’ in Fig. 1b). For safety and conservation reasons, the National Park Service prefers to identify the cave by number instead of providing its name or exact location. The cave contains an ice block that appears to be a remnant of a much larger (~ 1000 m^3^) congelation ice deposit that once filled most of the cave’s deepest section (see Supplementary Information S4). Its origin relates to a particular, seasonal ventilation that occurs in single entrance caves with descending passages (Fig. 1c). The denser winter cold surface air sinks along the cave floor and forces out the more buoyant warmer air at the ceiling level, causing the cave environment to become undercooled^21^. Due to the steep sides of the cave entrance, during summer, sunlight shines and warms-up only the air close to the entrance for a few hours. Thus, the surface warm air cannot penetrate far inside the cave to replace the colder air trapped during winter. As a result, air ventilation is restricted to the entrance zone and completely ceases in the interior where cave temperatures remain at, or below 0ºC (Fig. 1c). Under these particular settings, freezing of rainwater and snowmelt water percolating from surface through the basalt fissures builds perennial ice accumulations (floor ice and mounds) between late autumn and early winter and/or in the early spring. Thus, wet-cold conditions are ideal for higher ice accumulation rates. Wet-warm conditions can impact deposition of ice in two different ways: (1) if weather remains hot and rainy throughout summer and autumn, infiltration of warmer seepage water after occasional rainfalls causes some melting, which usually removes a thin layer from the top of the ice block, and (2) if winter and spring experience mild climatic conditions (wet and warmer than usually), drip water will be available and more cave ice will form. Under prevailing drier conditions (either dry-cold or dry-hot), ice accumulation ceases since no (or very little) infiltration water reaches the cave.

Unusual when compared with other caves hosting ice deposits, is the presence in Cave 29 of large amounts of charcoal and partly burnt wood (locally exceeding 30–40 cm in thickness; see Supplementary Fig. S2), charred wood, and ash deposits. These deposits cover the floor and breakdown boulders throughout the cave, but especially from the mid-passage constriction to the ice block where the core was taken and beyond into the back of the lava tube (Fig. 1b).

### Ice core stratigraphy and charcoal samples

**Figure 2:**
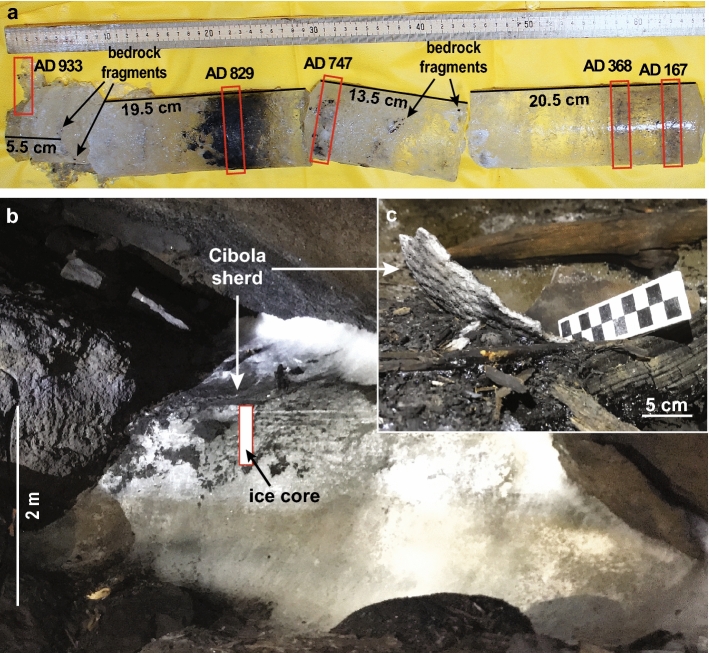
The ice coring site in Cave 29. (**a**) View of the ice core and placement of the radiocarbon samples (red rectangles). (**b**) The ice block showing the coring site and the position of the corrugated Cibola Gray Ware. (**c**) Close-up view of Cibola sherd next to charcoal fragments. Photos by B. P. Onac.

A 59-cm long core was drilled out from an ice deposit located in an alcove of the main passage towards the end of Cave 29 (Figs. 1b, 2a,b). Overall, the core exhibits a certain degree of layering due to incremental buildup of transparent and milky ice, but the most obvious stratigraphic feature is the occurrence of charcoal concentrations at various depths throughout the core and some soot-rich horizons (Fig. 2a). Five radiocarbon ages obtained on charred material recovered from the core suggest that the ice likely accumulated sometime prior to ~ AD 167 ± 29 and until at least ~ AD 933 ± 23 (Table 1).

**Table 1:** Radiocarbon ages and the calibrated years AD obtained on charcoal from Cave 29.

Depth from top (cm)	Radiocarbon age (yrs BP)	2σ calibrated age range yrs AD (probability %)	Date used (cal yr AD)
1.5	1119 ± 21	**888—981 (100)** 778—791 (3.1)	933 ± 23
1.5 *bis*	1130 ± 30	805—842 (5.9) **860—988 (91)**	923 ± 18
19	1201 ± 20	**770—886 (100)**	829 ± 29
27	1239 ± 22	789—871 (29.2) **687—779 (70.8)**	747 ± 23
50	1677 ± 21	265—271 (1.6) **331—415 (98.4)**	368 ± 21
55	1849 ± 25	86—109 (7.5) **117—235 (92.5)**	167 ± 29
55 *bis*	1833 ± 30	86—110 (4.2) **116—248 (95.8)**	179 ± 30
CS-1	940 ± 30	**1025—1160 (100)** 1048—1086 (12.2)	1097 ± 30
CF-1	863 ± 30	1123—1137 (2.9) **1149—1255 (84.9)**	1182 ± 30
CF-2	500 ± 30	1334—1336 (0.4) **1398—1448 (99.6)**	1424 ± 30

Bold range includes the *y*-intercept and has the highest probability distribution. The samples collected from within the corrugated Cibola Gray Ware sherd (CS-1) and cave floor (CF-1 and CF-2) along with two other replicates (labeled *bis*) were measured at Beta Analytic Radiocarbon Dating Laboratory.

Lying on top of the ice block next to the coring site, a large (25 × 17 cm) fragment of corrugated pottery was exposed as ice melting accelerated over the past decade (Fig. 2c). It was identified as Cibola Gray Ware, a type of ceramic that had a broad range of use in the area beginning with AD 900s^23^. Its presence is consistent with the settlement pattern for the Cibola area of Ancestral Puebloan sedentary agriculturalists residing in aboveground pueblos as residences^17,24^. A charcoal residue sample from within the sherd (CS-1) returned an age of AD 1097 ± 30. Two other pieces of charred pine tree needles (CF-1 and CF-2) collected from the cave floor at 15 and 17 m from the coring site (Fig. 1b) suggest burning occurred at AD 1182 ± 30 and AD 1424 ± 30, respectively (Table 1).

## Discussion

### Paleoclimatic and archeological context of Cave 29′s charcoal

**Figure 3:**
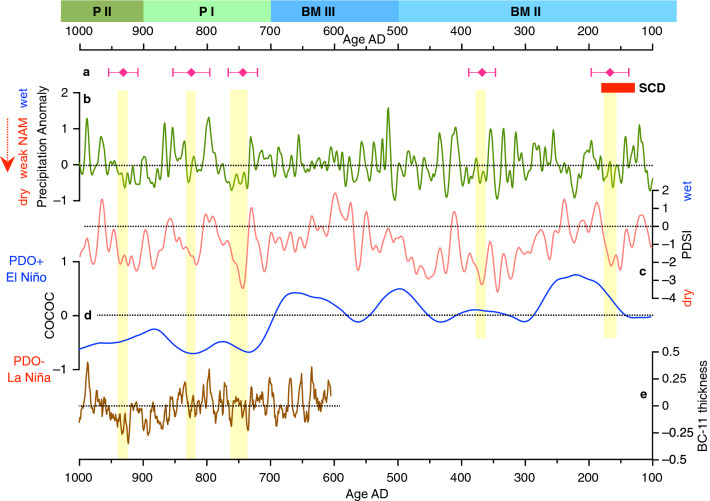
Drought episodes in the Southwest. (**a**) Radiocarbon dated charcoal samples from ELMA ice core. SCD is the Second Century Drought. (**b**) July precipitation reconstruction using ELMA latewood width chronology^15^. (**c**) PDSI^25^. (**d**) Variability of the composite coccolith proxy in the Santa Barbara Basin^40^. (**e**) Normalized band thickness in stalagmite BC-11^34^. BM and P refer to Basketmaker I-III and Pueblo I-II periods of the Pecos classification^1^, respectively.

The timing of all charcoal horizons (Fig. 3a) overlaps intervals with below-average summer rainfall as reconstructed by^15^ using latewood tree-rings from ELMA (Fig. 3b). They also correspond to drier periods as depicted in the continental-wide tree-ring based summer season Palmer Drought Severity Index (PDSI^25^; Fig. 3c), which is considered a measure of moisture availability to trees (tree-ring width increases with precipitation). The lower two charcoal levels in the ice core dated to AD 167 ± 29 and AD 368 ± 21 overlap a broad peak (~ AD 150 to 400) in charcoal influx in Kirman Lake, California^26^, interpreted to reflect aridity over a period with cool conditions in the eastern equatorial Pacific (La Niña state). In the Southwest, the effects of El Niño are synergistically enhanced during highly positive phases of Pacific Decadal Oscillation (PDO; Fig. 3d), bringing above-average Pacific-sourced winter moisture surplus^27^. Such periods with extreme winter precipitation in this region tend to be followed by summer-derived moisture deficit conditions, as persistent spring snow cover would delay the onset of the North American Monsoon (NAM)^15^. Thus, it is possible that a combination of multi-year La Niña-related winter precipitation deficits amplified by a negative state of PDO, and variation in summer monsoon patterns could better explain some of the persistent drought periods over the Southwest^26,28^. In fact, this scenario is consistent with results that associate the Medieval Warm Period drought (AD 800–1300) with persistent La Niña-like conditions and negative PDO^29,30^.

The age of the first charcoal horizon (AD 167) falls within the so-called Second Century Drought (SCD; Fig. 3a), a severe decades-long arid period (AD 122–172), first document in a tree-ring record from the Rocky Mountains^31^ and later recognized in the tree-ring record just west of ELMA^16^. This event is coincident with a significant positive temperature anomaly in the Northern Hemisphere and a period of high solar irradiance^32^. The SCD slowed down noticeably the growth of stalagmites from caves in the Guadalupe Mountains (Fig. 3e)^33,34^ and probably caused the hydrologic drought #6 in the Pyramid Lake (Nevada) sediment δ^18^O record^35^. A series of other sites across the central Great Basin and the midcontinental US also document a period of aridity between ~ AD 20–300^36^. Although it has been claimed SCD may have been equal if not more severe than the Medieval megadrought^31^, the total amount of charcoal particles found in our ice core corresponding to this interval is very small. This does not necessarily imply the drought was less severe than others in the record, but it likely shows that there was less human activity in this cave and perhaps more in adjacent lava tubes.

The second charcoal level dated at AD 368 falls within a multidecadal period of increased aridity (drought #8) in the Summerville bristlecone record^31^ and can be tentatively correlated (within the age uncertainty of our sample), with one of the drought events that occurred between AD 350 and 400 in west-central New Mexico^16^. These lower two charcoal and soot-rich layers correspond with early agricultural adaptations of the Basketmaker II (BM II) period, which in the Southwest is associated with the shift from foraging and residential mobility to agricultural dependence, the development of pithouse villages and seasonal if not year-round sedentism^37^. Although few in ELMA, BM II sites are well-documented regionally in the Zuni area^17,38^ (see Fig. 1a).

Since several regional records indicate enhanced aridity around AD 167 and AD 368^16,26,31^, it is tempting to associate the presence of the lower two horizons with micro-charcoal pieces in our core with anthropogenic ice melting practices, rather than wildfires. It is conceivable that the Ancestral Puebloan, who probably knocked down (by hand or with ground stone implements) ice stalactites and stalagmites accumulated near the cave entrance and carried them outside, moved to melt ice deeper in the cave when the need for water increased. Alternatively, the ice could have receded due to continual usage. Altogether, this supports our interpretation that minor BM II communities having temporary or seasonal camps nearby Cave 29 conducted small-scale melting ice activities for securing water for drinking and/or ceremonial purposes over these time periods. It is also expected that the water resources hidden in some of the lava tubes throughout ELMA were known to the Ancestral Puebloans who used a well-established network of trails that date back to prehistory (e.g., Cerritos de Jaspe^7,18^) to reach to these resources (Supplementary Fig. S1).

Corroborating the lack of charcoal at ELMA between ~ AD 400 and 700 with available paleoclimatic evidence, suggest generally wetter/colder conditions in parts of the Southwest, with less frequent or no summer wildfires^26,33,39^. Lowering of the mean annual temperature coupled with increased precipitation, caused an expansion of the cave ice that may have obstructed the narrow median passage, hence the access to the main ice deposit. Such instances are known in ELMA where entrances to some lava tubes remained ice plugged until recently^18^. If this was the case for Cave 29, no soot or charred material is expected to reach the coring site even if the Ancestral Puebloan continued to harvest ice accumulated in the first half of the cave. Due to an increase in drinking water availability from snowmelt and wetter summers, probably, cave ice melting came to be a backup option. It is equally possible that overall wetter conditions increasing water tables and water flow in the low lands, kept Basketmaker II and III groups at lower elevations in the Zuni River Valley during this interval, where they farmed using irrigation canals to cultivate maize^38^.

The upper 30 cm of our ice record spans the time period from AD 700 to ~ AD 950. It includes three charcoal horizons (Fig. 2a and Table 1), all of which happened at times of low summer precipitation in the ELMA tree-ring-derived record^15^ (Fig. 3b), some of the most negative values of PDSI^25^ (Fig. 3c), thinner calcite layers in stalagmite BC-11^34^ (SE New Mexico; Fig. 3e), and a megadrought affected the Mexico highlands^41^. Comparing the charcoal occurrence in Cave 29 with other records, we observed that soon after ~ AD 700 the Northern Hemisphere temperature begins to increase, the tropical Pacific experienced long periods of La Niña-like state^27^, which coupled with dominant negative-phase PDO^40^ (Fig. 3d), higher sea-surface temperatures in the North Atlantic^42^, and below average temperatures for the western US, mark the onset of a prolonged interval of increased aridity that culminated with the Medieval megadrought^30,42^. In addition, the finding by Stahle et al.^15^ indicates that years to decades of drier-than-average monsoon season (Fig. 3b; weak NAM) occurred during this time interval, prompting concurrent robust and widespread droughts across the Southwest^30,33,39^. Higher abundance and larger charcoal particles in the ice core following the combustion event from AD 747 ± 23 support an interpretation of extended periods of soil water deficit in ELMA during the summer. A severe drought episode is documented by the thickest charcoal horizon (~ 3 cm) in the ice core dated at ~ AD 829 ± 29. This marks the first indisputable, large-scale melting activities beyond the narrow median section of the cave.

These two charcoal layers fall within the P I period, when ELMA saw modest increases in population characterized by dispersed settlement and continued use of pithouse structures along with early surface architecture including pueblos of masonry and jacal^17^. The combustion event occurring ~ AD 829 could indicate an increased aridity event that forced the Pueblo I people in the ELMA region to melt more cave ice over a longer period of time. The presence of this charcoal layer alone, however, cannot be taken as evidence of a larger community in the region. This is because surface site frequency around Cave 29 remains relatively low and no long-term residential sites have been documented in the area, except for small clusters of stacked basalt rock enclosures around the cave entrance, possibly associated with hunting (e.g., deer and bighorn) or other short-term resource-focused occupations, including water collection^18^.

The drought conditions that persisted throughout the Medieval Warm Period increased the demand for water, pushing Pueblo II and III communities in ELMA or travelers along some of the trail routes (trade, pilgrimage, or migration) to undertake sustained exploitation of ice in Cave 29). This is revealed by the artifact assemblage documented by Powers and Orcutt^17^ at Cave 29 and two other surface sites nearby, which is dominated by ceramics, mostly jar sherds dating to the P II period when the use of the lava fields and flow margins increased significantly^11^. In addition, four other ^14^C-dated charcoal samples, one from the uppermost horizon in the ice core (AD 933), one from inside the Cibola sherd exposed on top of the ice deposit (AD 1097), and two from the cave floor deposit (Table 1), further suggest ice harvesting continued through P II and P III. Large charcoal fragments and ashed plant material (pine tree needles and twigs) accumulated in the rear of the cave indicate that they could not have been transported from afar into the cave by water or wind but were carried in and combusted, thus accumulated locally.

We interpret the lack of charcoal younger than AD 950 in the ice core, but its abundance on the cave floor to suggest that the uppermost part of the cored ice deposit melted during the Medieval era megadrought. The age of the charcoal residue collected from the interior of the ceramic sherd, AD 1097, indicates a Pueblo II period ice melting activity. The in-situ discovery of the sherd, its position in relation to the top of the ice deposit, and the presence of charcoal residue coating its interior surface provides insights on the method employed by pre-contact Puebloan people (since the P I period) to obtain water from the cave ice.

The last documented use of fire to melt ice in Cave 29 comes from well preserved charred material scattered around and between breakdown blocks some 27 m from the end of the gallery. The radiocarbon age suggests the combustion episode dates to ~ AD 1424 and is coincident with a hydrologic drought that struck the Southwest as revealed by extended low flows in the Colorado River Basin^43^. It could be that this fire event involved a small group of Ancestral Puebloans hunters or travelers of the P IV period on pilgrimage to collect water at Cave 29 or stopping at the cave in transit between regions along the trail routes across the lava fields.

We speculate that during the Little Ice Age (~ AD 1500–1850) the gallery beyond the mid narrow passage was again sealed with ice since no younger charred material was found so far. It is plausible that during this period new ice accumulated throughout the deeper part of the cave, including in the proximity of the coring site. This is probably when the Cibola corrugated sherd with charcoal residue from the P II period was completely covered by ice. The accelerated melting of ice documented over the past decade, exposed the sherd.

### Water harvesting and handling

Whether the Ancestral Puebloans are responsible for burning wood inside the cave almost 2000 years ago in order to obtain drinking water, or the charred material originating from wildfires was transported underground by water and/or wind could be a point of debate. Because the radiocarbon-dated horizons contain a mixture of fine (soot) and millimeter-size angular charred particles (Supplementary Fig. S3), theoretically, both scenarios are possible. Charcoal from torches (in deeper caves) and in hearths (in surface tubes) were documented in caves that are presently ice-free or in which evidence of past ice deposits no longer exist. When the presence of charcoal is related to the use of torches (pilgrimage activities, visits, etc.), ash and charcoal fragments appear as isolated scatters. In caves throughout ELMA where ice harvesting occurred, abundant ash and charcoal deposits blanket the cave floor and several millimeters thick soot deposits cover the walls and ceiling^7,19^. Corroborating this information with the total absence of charcoal between the entrance and narrow median section of Cave 29 (Fig. 1b), suggest that local combustion events occurred near the coring site. Regarding the presence of soot in the ice core, one can argue it entered through the fairly large cave entrance during major wildfires that are common in this area (see Supplementary Information S3). However, this hypothesis is refuted by the particular air circulation within this cave that ceases during the warm season. Any forest fire related soot can reach the inner part of the cave only if the event happens late in fall when outside temperature drops overnight below 0ºC and the cold air convection cell is activated (Fig. 1c).

An interesting question regarding the use of fire by Ancestral Puebloans, is how they handled the smoke in the rear part of the cave, knowing that the summer air circulation model within Cave 29 implies no exchange with surface. However, hot air and smoke emitted during combustion events in the inner part of the cave will always rise and generate an outward flow along the ceiling^44^. Therefore, if small fires were used, the smoke would efficiently evacuate allowing people to remain in the cave to collect melt water and tend the fire. Alternatively, if the combustion events involved larger fires deeper in the cave, they probably had to wait outside for as long as smoke continued to exit the cave. In support of this option, we note the existence of physical alterations on the cave floor (excavations, though primarily in the front passage of the cave) to capture water perhaps from larger and smokier unattended combustions. Smaller fires would allow better control of the melting and collection process providing cleaner water in smaller quantities than unattended larger fires that produce water at a faster rate with greater potential for contamination from charcoal and ash. Both scenarios point toward ways to capture melt water from perennial cave ice.

The presence of pots or even bowl-shaped sherds (including the corrugated Cibola Gray Ware fragment next to the coring site) could have served used as a platform to support small fires or coals and allow for water collection while melting ice. The curvature of the sherd acted as a container for the heat generated by fire or coals that in turn warmed the sherd and transferred that heat melting the ice, which was collected in vessels or from the cave floor. The melt water may have been directed to hand-held ceramic or pitch-coated basketry receptacles on the cave floor using a variety of methods such as branches or timbers cut along their axis. A timber with a trough-like groove cut along its axis is documented in another lava tube cave in ELMA, and though it likely dates to the historic era, its purpose appears to be associated with melting ice^18^. While not direct proof, the use of grooved or troughed wood to direct water supports the scenario of tending small fires and collecting water while in the cave.

Surface archeological sites in the vicinity of the cave are dispersed and contain little evidence of year-round occupation tending instead to low diversity of features and material culture and lacking substantial architecture and middens. Population centers throughout ELMA’s Puebloan sequence lie along the margins of the lava fields 25 km east of Cave 29 and in the Cerro de Jaspe’ area 8 km northeast of the cave^17,18^. Windes^18^ and others have noted the distinct east–west trail that passes by Cave 29 and through the stacked basalt enclosure site near the cave entry. This is one among a network of trails that crisscross ELMA’s lava fields including the Acoma-Zuni trail, a well-known travel corridor that bisects the northern half of ELMA. Many of these trails link caves with caves and/or pre-contact pueblo sites along the flow margins and in kipukas in the lava flows^17^ (Supplementary Fig. S1). Such trails may have conducted Ancestral Puebloans the considerable distance from residential bases on the east and north sides of the lava fields to Cave 29 and other ice caves during pilgrimages to collect melt ice water, as travelers moving between the Acoma and Zuni areas or hunters seeking game at the cave or near it^6,7,18^.

An estimated 1.000.000 L of water would have been available from the ice in Cave 29 making it a reliable and long-term water source for the area that could have supported opportunistic as well as frequent utilization of the resource. Knowledge of the location and reliability of this water resource is suggested by the clusters of small, stacked basalt structures near the cave (and near other caves and crevasses where ice forms in ELMA’s lava flows), suggesting that the location could have served as a stopover for travelers and hunters or as an anchor point for logistical foragers targeting seasonally available resources in the area^17,18^. Ethnographic studies in the Cibola region have identified cave ice as a target resource for ritual activity, pilgrimage and for medicine societies seeking pure water from the ice for ceremonial and medicinal use^6,7^.

## Conclusion

This study robustly characterizes five drought periods, during which Ancestral Puebloans entered Cave 29 and other lava tubes to harvest ice. We suggest that prior to Pueblo I people who left behind recognizable artifacts within ELMA, small groups of Basketmaker II and III utilized the cave seasonally for melt water for domestic purposes, while hunting or exploiting other resources but lived in communities in low land valleys of the Zuni area. Based on a variety of evidence for burning in archeological context, the fire-aided ice melting activities carried out during P I-III increased notably throughout the Medieval Warm Period. Physical modifications to the floor between the cave entrance and the narrow mid-passage are testament to water procurement activities at many times in the human history of the cave. The seasonal melting of ice near cave entrances leaves temporary shallow pools of water that would have been accessible to Ancestral Puebloans who surely would have taken advantage of this readily available water for drinking. When ice was absent or retreated in warmer, dryer periods and/or was harvested in this part of the lava tube, Ancestral Puebloans would have worked their way to the back of the cave, melting ice and leaving charcoal and ash deposits along the way. The process may have taken decades and perhaps repeated many times depending on the ice volume fluctuation. They certainly used the water from melted ice for drinking, but ceremonial and medicinal use cannot be excluded, since ice was considered purer and more desirable for harvesting for such purposes^7^. For example, lava tube caves were significant destinations for religious pilgrimages and use of these areas to help keep abreast of what plant, animal, and mineral resources were available^6,18^. Since Cave 29 sits on well-established trail routes crisscrossing ELMA, it is possible that the melted ice could also have been used during trading missions^6^ or accessed by migrants passing by the lava tube. Regardless, the timing of the charcoal fragments preserved within the cave ice deposit discussed here sheds light on one of many human–environment interactions documented in ELMA in a context highly susceptible to the influence of climate change. It also highlights the importance of an increasingly threatened source of paleoenvironmental and archeological evidence. The melting of cave ice under current climate conditions will result in the loss of ancient climate data. As such, this resource must be further examined in the near future before it completely vanishes.

## Methods

### Ice block, ice core acquisition, and charcoal samples

An estimated ~ 1000 m^3^ ice block in Cave 29 once filled-up much of the large gallery beyond a constriction that occurs halfway between the entrance and cave’s farthest point (Supplementary Information S4). Multiple lines of evidence, including cryogenic calcite dust marks along the walls and charred material support this assumption. Ember fragments of various sizes projected from the fires used by Ancestral Puebloans to melt ice, accumulated on the ice surface. These indicators of anthropogenic fires were subsequently covered by the newly formed ice. In remote locations, unaffected by ice melting activities, such as the coring site, they remained trapped in the ice. In addition, beyond the constriction and adjacent to where the core was drilled, the cave floor is covered for ~ 45 m with charcoal deposits that vary between 15 and 30 cm in thickness. The coring site represents a remnant of the ice block preserved in a side passage towards the end of the cave (Supplementary Fig. S4). It contains multiple soot and charcoal-rich layers as well as minute fragments of bedrock that fell from the ceiling (Fig. 2a). Photographs taken between the mid-1980s and late 2010 reveal that ice had reduced about 25 cm at this location. An additional ~ 5 cm of melting occurred since then, uncovering the corrugated Cibola Gray Ware sherd. In April 2017, a 59 cm long core (5 cm diameter) was drilled next to this artifact, using a Bosch handheld corer. The recovered ice core was photographed, described, and cut into disks with a diameter of 5 cm and a thickness of 1 cm while in the cave. These were later melted at room temperature and the solid residue was extracted on Sartorius borosilicate glass microfiber filter disks for radiocarbon dating.

### Radiocarbon dating of charcoal

Seven twig charcoal fragments and filtered organic matter recovered from the ice core and a sample of charred pine needles (CF-1) were sent to the RoAMS Laboratory at the National Institute for Physics and Nuclear Engineering in Romania, for radiocarbon dating using a 1 MV Tandetron accelerator mass spectrometer^45^. Although charcoal was recovered from depths of 7.5 and 33 cm in the ice core, not enough graphite was produced to generate ages. Two additional samples were measured using the AMS method at the Beta Analytic Radiocarbon Dating Laboratory. CF-1 represents charred pine tree needles from ~ 3 cm below the surface of the thick charcoal deposit accumulated on the cave floor, 17 m away from the ice coring site. The second additional sample that was not associated with the ice core, is CS-1, a charcoal residue collected from the interior of the corrugated Cibola Gray Ware sherd exposed by melting of ice in Cave 29 next to the coring site. Calibration of the ^14^C ages was performed with BetaCal3.21 software using the age distributions obtained with the IntCal13 curve^46^. For inter-laboratory comparison purposes, the charcoal samples from 1.5 and 55 cm in depth were dated in both AMS facilities (Table 1). The results between RoAMS and Beta Analytic laboratories are indistinguishable at a 95% confidence level.

## Supplementary information

Supplementary Information.

Supplementary Figure S1.

Supplementary Figure S2.

Supplementary Figure S3.

Supplementary Figure S4.

Supplementary Figure S5.

Supplementary Figure S6.

## Data Availability

All data needed to support the conclusions of this paper are included in the main text.
