# Supplementary material for: Late Holocene droughts and cave ice harvesting by Ancestral Puebloans
Source: Sci Rep. 2020 Nov 18;10:20131. doi: 10.1038/s41598-020-76988-1 (PMC7674407; doi:10.1038/s41598-020-76988-1)
Supplement: Supplementary file 1 — Supplementary Information. [file 41598_2020_76988_MOESM1_ESM.docx]

**Late Holocene droughts and cave ice harvesting by Ancestral Puebloans**

Bogdan P. Onac^1,2,*^, Steven M. Baumann^3^, Dylan S. Parmenter^4^, Eric Weaver^3^, Tiberiu B. Sava^5^

^1^School of Geosciences, University of South Florida, Tampa, FL 33620, USA

^2^Emil G. Racoviță Institute, Babeș-Bolyai University, 400006 Cluj-Napoca, Romania

^3^National Park Service, El Malpais and El Morro National Monuments, Grants, NM 87020, USA

^4^Department of Earth Sciences, University of Minnesota, Minneapolis, MN 55455, USA

^5^Horia Hulubei National Institute for Physics and Nuclear Engineering, 077125 Măgurele, Romania

*Correspondence and requests for materials should be addressed to BPO (bonac@usf.edu)

**Supplementary Information**

**S1. Archeological background.** Located in the heart of the Western Pueblo world, El Malpais National Monument’s (hereafter ELMA) archeological landscape is typical of the Four Corners region with distinctive Puebolan period sites including the twelfth century Chaco style great house and great kiva site of Las Ventanas. To the east lies the Cebollita Mesa area and the Pueblo of Acoma beyond. To the west are the lava fields of ELMA. This region is part of the ancestral homeland of the Acoma people and is culturally significant for archaeological resources and wild and scenic values^1^. The Las Ventanas community includes the great house and great kiva and a dispersed smaller pueblo sites dating to the Pueblo II period (AD 900-1150) all positioned along the top of Putney Mesa overlooking the lava fields to the west. Cebollita Mesa is the prominent landform and high point for the region. Early research in this area by^1^ and^2,3^ included Putney Mesa and laid the foundation for the cultural-chronological sequence of the area. Subsequent cultural resource management studies of the area helped refine the cultural sequence and are relevant to the understanding of subsistence-settlement patterns in ELMA. The mesa tops and canyons bordering the eastern edge of ELMA’s lava flows were at the center of the region’s emergent 9th through 13th century Pre-Contact Puebloan communities whose territory extended far into ELMA’s lava flow landscape.

Human occupation of the ELMA - Cebollita Mesa region extends back to the Paleoindian period based on sparse but clear evidence from material culture (likely Folsom) in the Armijo Canyon area^4^. Subsequent hunter-gather adaptations from the early through late Archaic period (8,000-1,500 B.C.) also are documented by material culture found on the Cretaceous-age sandstone mesas and ridges of Cebollita and Putney mesas along ELMA east side^1,5^ as well as the older lava fields and remnant Zuni Mountain steptoes and *kipukas* on ELMA’s west side^6,7^). Cultural material evidence of these early occupations in the lava fields is rather more circumstantial, however, represented by lithic materials most frequently associated with later Puebloan occupations. Evidence of human activity on the McCartys flow and especially the earlier, tube-bearing lava flows during the Puebloan cultural sequence is extensive^5,8^.

**S2. Geology, present-day climate, and forest fires.** Eruptions in the Zuni-Bandera volcanic field began as early as 700.000 years ago, but in ELMA, the basalt flows are younger than 100.000 years^9,10^. Both aa (jagged and very broken) and pahoehoe (smooth and ropy) lava type flows exist, but only the latter one supports the formation of lava tubes^11^; Cave 29 is in Bandera Flow (~11.000 years)^12^. El Malpais’ youngest lava flow is from the McCartys cinder cone and was active 3,900 years ago, effectively covering probable evidence of earlier human use of older lava^9,13^ (Supplementary Fig. S1). From a geomorphological point of view, ELMA is a high elevation (2100 to 2600 m) desert environment. The mean annual temperature in ELMA is 10.3ºC (-36º to +41ºC), typical for a continental climate. Precipitation averages 218 mm, with 35-40% occurring as monsoonal rainfall between July and August and 37% falls in winter (October through March) when low-pressure systems moving from west to east across the Southwest, coalesce with moisture from the Pacific Ocean or the Gulf of Mexico^13,14^. Unless localized thunderstorms deliver higher than normal rainfall, the summer precipitations are hydrologically less important because much of the rain evaporates before it percolates into the subsurface. Winter precipitation falls primarily as snow that melts in early spring and infiltrates into the ground. The vegetation in the area comprises ponderosa and piñon-juniper woodland and savanna, whereas the understory is dominated by native short prairie grasses interspersed with cacti^14^ (Supplementary Fig. S5) Depending on their size and age, which among others control soil development, each lava flow within ELMA supports characteristic vegetation assemblages. Chances for natural fire ignition (drought, lightning, etc.) and spread are specific to each individual plant habitats (e.g., ancient or young basal flow, cinder cones, kipukas), thus, frequency, proportion, and intensity of wildfires are not all the same throughout the park^15^. The cold season is not the wildfire season that typically begins during mid-summer monsoons and sometimes persists through the end of September. The collapsed lava tube that forms the trench in front of the cave is virtually free of vegetation (other than wet moss garden). There is little fuel to carry fire to the cave entrance. Though heavy fuels could fall in the trench during wildfire, there is no evidence around the cave entrance.

Reconstructions of wildfires events using tree-ring data collected in ELMA cover only the period since AD 1350 and indicate that prior to 1880 the majority of fires occurred approximately once five to eight years^16,17^. In terms of seasonality, the data available between 1600 and 1991 suggest that most wildfires occurred in the early part of the growing season (April to June), except for the 1740–1840 interval, which was characterized by late-season fires^15^. The records compiled for the American Southwest suggest century-long climate forcing of wildfire events, especially during dry years that are often associated with La Niña^18,19^.

**S3. Lava tubes and ice accumulation in ELMA.** Lava tubes are found mainly on ELMA’s west side in the El Calderon, Hoya de Cibola, Twin Craters, and Bandera lava fields^10^. Master tubes and distributary tubes were primary conduits for lava along the length and breadth of the flows creating numerous passages^11^. Lava tubes range from small and constricted surface tubes to long, deep caverns. Sections of deeper tubes are cold traps where temperature and relative humidity conditions remain constant year around and percolating water (from rain or snowmelt) is preserved in accumulations of perennial ice in the freezing zone^20-22^. Seasonal ice in ELMA occurs near the cave entrances as frozen pools or sheets on cave floors, ice walls, ice stalagmites and stalactites, and as ribbons of ice clinging to walls or breakdown. This ice represents a source of water that lasts until early summer when sunlight shines through the cave entrance and promotes ice melting. Instead perennial ice deposits, which accumulate further inside the lava tubes provide a year-round water source. Such lava tubes are thought to have been an important source of domestic water for the Puebloans in the ELMA area^5,23^. Worth noting is that like the seasonal ice formations and the ebb and flow of the perennial ice is linked with the availability of percolation water entering caves. For example, in 1988, the perennial ice in Cave 23 (in the vicinity of Cave 29) grew large enough to block the main passage of the cave, receding to a small deposit and ice ponds 20 years later^23,24^.

**S4. Cave ice volume estimation.** Generally, lava tubes in ELMA have a rather classic internal morphology (hollow passages with circular, elliptic, or oval cross-sections and localized breakdown piles), which allow three-dimensional (3D) volume calculation based on spatial partitioning, e.g., using voxel (volume elements like cylinders, oval, or rectangles). The result is a discrete image of the volume model. To calculate the volume of Cave 29 beyond the constriction (Fig. 1b), we divided this part of the cave into three oval tubes for which the length (L), width (W), and height (H) were obtained from the cave map generated by using a high-resolution terrestrial laser scanning technology (Supplementary Table S1). The volume of each tube is calculated by multiplying the area (A) of the discorectangle (i.e., a geometric shape similar to a stadium, which in fact is a rectangle with equal semicircles on both ends) by the length (L) (Equation 1 in Supplementary Fig. S6). Using the data from cave mapping in Equation 1, a conservative total volume (V_tot_) of 1975 m^3^ was computed by summing up the volume of each oval tube. Next, we estimated how much ice would fit into this cavern. From monitoring studies conducted in other ice caves around the world^25-28^, it is known that regardless of ventilation type, caves can only host a limited amount of ice and cannot be filled completely, unless they are vertical shafts. As ice volume progressively increases, the available space for surface-sourced cold air accumulation decreases, and the conditions in which new ice layers form become less and less favorable. Considering the morphology of cave passage beyond the constriction zone, which controls the flow of cold air towards the inner part of the cave and using the calcite bathtub line present on the walls as a marker for the highest level of ice (see Results), we inferred that the ice block had a maximum thickness of 3.02 m (i in Supplementary Fig. S6a). The volume occupied by ice (V_ice_) in each discorectangle was calculated using Equation 2 (see Supplementary Fig. S6). To do this, we first computed the volume of ice fill in the rectangle (V_ice-rectangle_) and in the cylinder (V_ice-cylinder_) assuming they were filled with 3 m of ice. The ice filled volume of a rectangle is length (L) times width (a) times ice thickness (i). To calculate the volume of ice that partly fills a horizontal cylinder, we used Equation 3 (Supplementary Fig. S6) in which we subtract from the total volume of the cylinder (V_cylinder_ = πr^2^L) the area of a circular segment (gray shaded part of the circle in Supplementary Fig. S6) that represents the air-filled portion of the tube. Adding the three values (Supplementary Table S1), a maximum V_ice_ of 1377 m^3^ was obtained. However, because passages are not perfect geometric shapes and few areas are covered by collapses, which could not be accounted for, we consider these calculations as estimates. Thus, a more conservative and realistic volume of ice would be ~1000 m^3^ that represents ~1.000.000 liters of water.

**Supplementary References**

1 Dittert, A. E., Jr. *The prehistoric population and architecture of the Cebolleta Mesa region, Central Western New Mexico* Ph.D. thesis, University of Arizona, (1959).

2 Ruppé, R. J. & Dittert, A. E., Jr. Archaeology of Cebolleta Mesa: A preliminary report. *El Palacio* **58**, 116-129 (1951).

3 Ruppé, R. J. & Dittert, A. E., Jr. Acoma Archaeology: A preliminary report of the final season in the Cebolleta Mesa Region, New Mexico. *El Palacio* **60**, 259-273 (1953).

4 Elyea, J., Hogan, P. & Wilson, C. D. The Armijo Canyon archeological survey. Prepared for the Bureau of Land Management, Rio Puerco District, Office of Contract Archeology. (University of New Mexico, Albuquerque, 1994).

5 Powers, P. R. & Orcutt, J. D. Vol. Intermountain Cultural Resources Management Professional Paper No. 70, 270 (National Park Service, Denver, Colorado, 2005).

6 Baumann, S. M. & Nurminem, A. Archaeological inventory of the Aqua Fria Meadows prescribed burnt unit. 18 (Technical Report, El Malpais National Monument, Grants, NM, 2009).

7 Reed, P. F., Myers, T. & Throgmorton, K. Archeological Inventory of Four Prescribed Burn Units (Bandera Flow, East Encerrito, Mesita Blanca, and West Cerro Bandera) on the El Malpais National Monument, New Mexico, 2007. (Technical Report No. 2008-001, Center for Desert Archaeology, Tucson, 2007).

8 Zedeño, M. N., Schrag-James, J. & Basaldu, R. C. Overview and Inventory of ethnographic resources for Petrified Forest National Park, El Malpais National Monument and National Conservation Area, and El Morro National Monument. (Unpublished report on file at the Bureau of Applied Research in Anthropology, Tucson, 2001).

9 Laughlin, A. W., Poths, J., Healey, H. A., Reneau, S. & WoldeGabriel, G. Dating of Quaternary basalts using the cosmogenic ^3^He and ^14^C methods with implications for excess ^40^Ar. *Geology* **22**, 135-138, doi:10.1130/0091-7613(1994)022<0135:DOQBUT>2.3.CO;2 (1994).

10 Polyak, V. J., Dillon, J. R., Asmerom, Y. & Onac, B. P. Chronology of young basalt flows from lava tube gypsum U-series ages. *Quaternary Geochronology* **59**, 101083, doi:https://doi.org/10.1016/j.quageo.2020.101083 (2020).

11 Rogers, B. W. & Mosch, C. J. in *Natural history of El Malpais National Monument* Vol. 156 (ed K. Mabery) 61-68 (New Mexico Bureau of Mines & Mineral Resources, 1997).

12 Laughlin, A. W. & WoldeGabriel, G. in *Natural history of El Malpais National Monument* Vol. 156 (ed K. Mabery) 25-29 (New Mexico Bureau of Mines & Mineral Resources, 1997).

13 Valentine-Darby, P. *et al.* El Malpais National Monument: Natural resource condition assessment. Natural Resource Report NPS/SCPN/NRR—2016/1301. Report No. NPS/SCPN/NRR—2016/1301, 367 (National Park Service, Fort Collins, Colorado, 2016).

14 Muldavin, E. *et al.* Vegetation Classification and Map: El Malpais National Monument. Natural Resource Technical Report NPS/SPCN/ NRTR–2013/803. Report No. NPS/SPCN/ NRTR–2013/803, 73 (National Park Service, Fort Collins, Colorado, 2013).

15 Grissino-Mayer, H. D. & Swetnam, T. W. in *Symposium on fire in wilderness and park management.* (eds J.K. Brown, R.W. Mutch, C.W. Sopoon, & R.H. Wakimoto) 195-200 (U.S. Deaprtment of Agriculture, Forest Service).

16 Grissino-Mayer, H. D. & Swetnam, T. W. in *Natural history of El Malpais National Monument* Vol. 156 (ed K. Mabery) 163-172 (New Mexico Bureau of Mines & Mineral Resources, 1997).

17 Lewis, D. B. *Fire regimes of kipuka forests in El Malpais National Monument, New Mexico* Master of Science thesis, University of Tennessee, (2003).

18 Swetnam, T. W. & Baisan, C. H. in *Fire and Climatic Change in Temperate Ecosystems of the Western Americas* (eds Thomas T. Veblen, William L. Baker, Gloria Montenegro, & Thomas W. Swetnam) 158-195 (Springer New York, 2003).

19 Grissino-Mayer, H. D. & Swetnam, T. W. Century scale climate forcing of fire regimes in the American Southwest. *The Holocene* **10**, 213-220, doi:10.1191/095968300668451235 (2000).

20 Dickfoss, P. V., Betancourt, J. L. & Thompson, L. G. in *Natural history of El Malpais National Monument* Vol. 156 (ed K. Mabery) 91-112 (New Mexico Bureau of Mines & Mineral Resources, 1997).

21 Parmenter, D. S. *Ice and guano deposits in El Malpais lava tubes: Potential paleoclimate archives for the Southwest United States* (Unpublished MS Thesis, University of South Florida, Tampa, 2018).

22 Ohata, T., Furukawa, T. & Higuchi, K. Glacioclimatological study of perennial ice in the Fuji Ice Cave, Japan. Part 1. Seasonal variation and mechanism of maintenance. *Arctic and Alpine Research* **26**, 227-237 (1994).

23 Windes, T. C. A bighorn sheep trap at El Malpais National Monument, New Mexico. *KIVA* **74**, 71-105 (2008).

24 Baumann, S. M. & Kendrick, J. W. Climate change and the deterioration of cultural resources: El Morro and El Malpais National Monuments in west-central New Mexico. 17-21 (National Park Service, Denver, 2010).

**Supplementary Fig. S1.** Map of the El Malpais National Monument showing boundaries of the major lava flows and part of the Ancestral Puebloans trail network. The numbers refer to Candelaria Ice Cave (1) and Cerritos de Jaspe/Pack (2) and Acoma–Zuni (3) trails. The figure was produced with ArcGIS Desktop v. 10.6 by ESRI (https://desktop.arcgis.com/en/) using the geodatabase on file at ELMA.


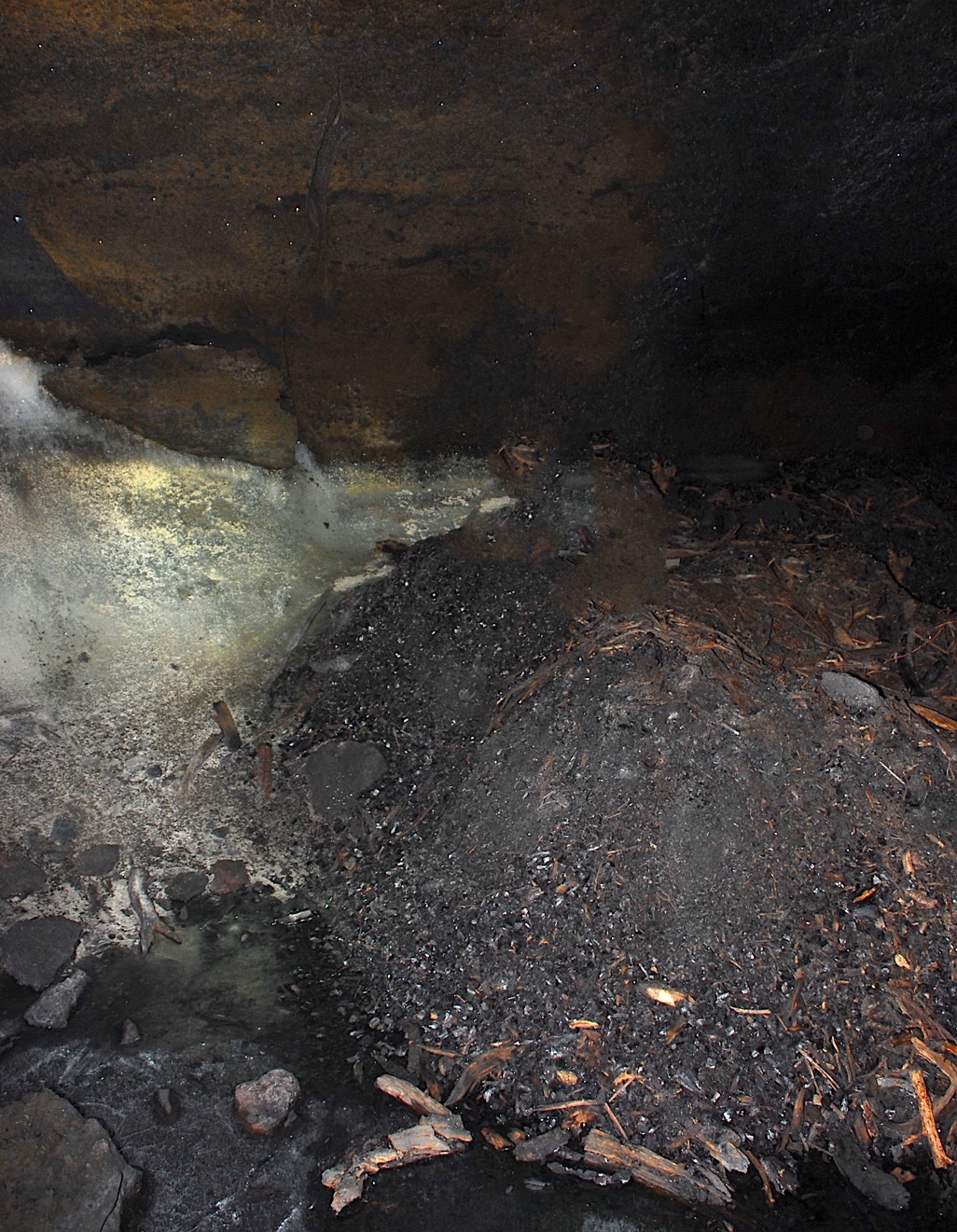


**15 cm**

Supplementary Fig. S2. View of charred material at the edge of the ice block in Cave 29 (Photo by B.P. Onac).

**Supplementary Fig. S3.** (**a**) Microphotograph of charcoal fragments from the ice core horizon dated to AD 829, taken with a Nikon SMZ 1500 binocular. (**b**) Secondary electron microphotograph of soot particles (bright white) in sample NIC 34 (AD 368) collected with a JEOL JSM 6490 scanning electron microscope. Photos by B.P. Onac.

**
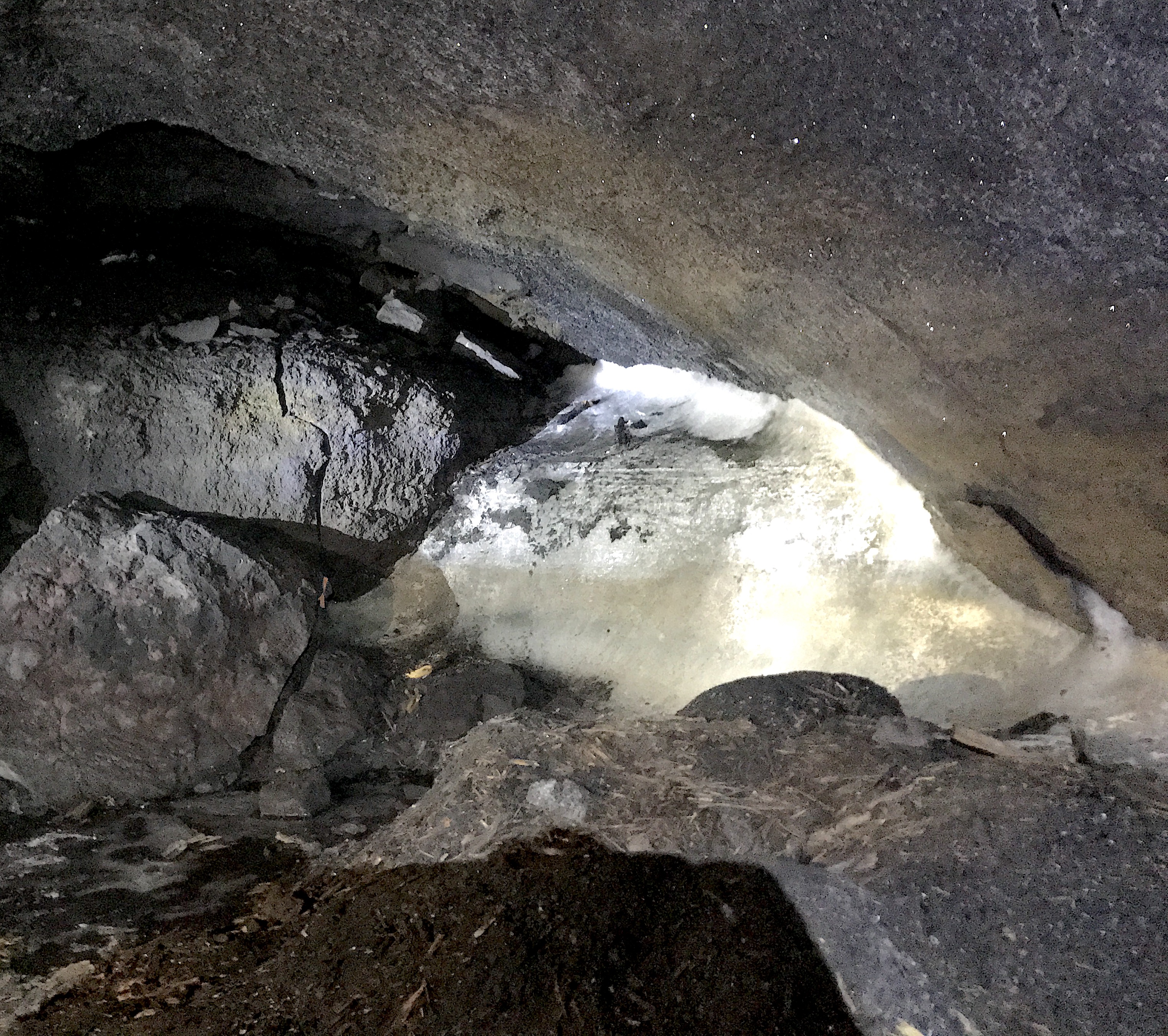
Supplementary Fig. S4.** Overview of the ice deposit (maximum height ~2.5 m) and the charcoal blanket covering the cave floor (foreground) (Photo by B.P. Onac).


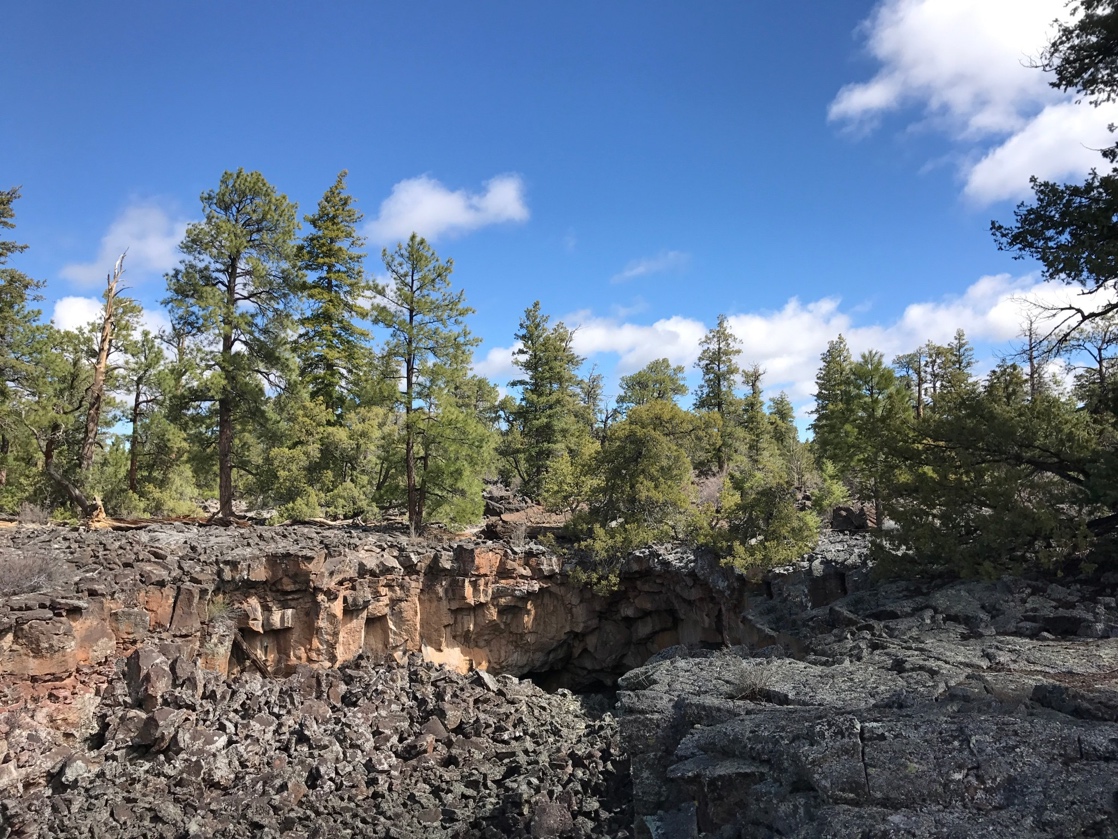


**Supplementary Fig. S5.** Landscape photography showing ponderosa pines growing on pahoehoe basalt flow and the collapsed section of a lava tube (Photo by B.P. Onac).

**Supplementary Fig. S6.** Elements of the discorectangle and a circular segment and the equations used to estimate the total volume and the volume of ice fill. The drawing was produced with Adobe Illustrator CC 2020 v. 24.2.3 (https://www.adobe.com/creativecloud.html).

**Supplementary Table S1.** Measurements used to estimate the ice volume in Cave 29.

| **Voxel** | **Length (m)** | **Width (m)** | **Height (m)** | **Volume**  **(m^3^)** | **Ice thickness (m)** | **Ice volume (m^3^)** |
| --- | --- | --- | --- | --- | --- | --- |
| Tube 1 | 40 | 8 | 4.5 | 1266 | 3 | 871 |
| Tube 2 | 20 | 7 | 4.5 | 543 | 3 | 375 |
| Tube 3 | 10 | 5 | 4 | 166 | 3 | 131 |
