# Supplementary figures and images for: Late Holocene droughts and cave ice harvesting by Ancestral Puebloans
Source: Sci Rep. 2020 Nov 18;10:20131. doi: 10.1038/s41598-020-76988-1 (PMC7674407; doi:10.1038/s41598-020-76988-1)

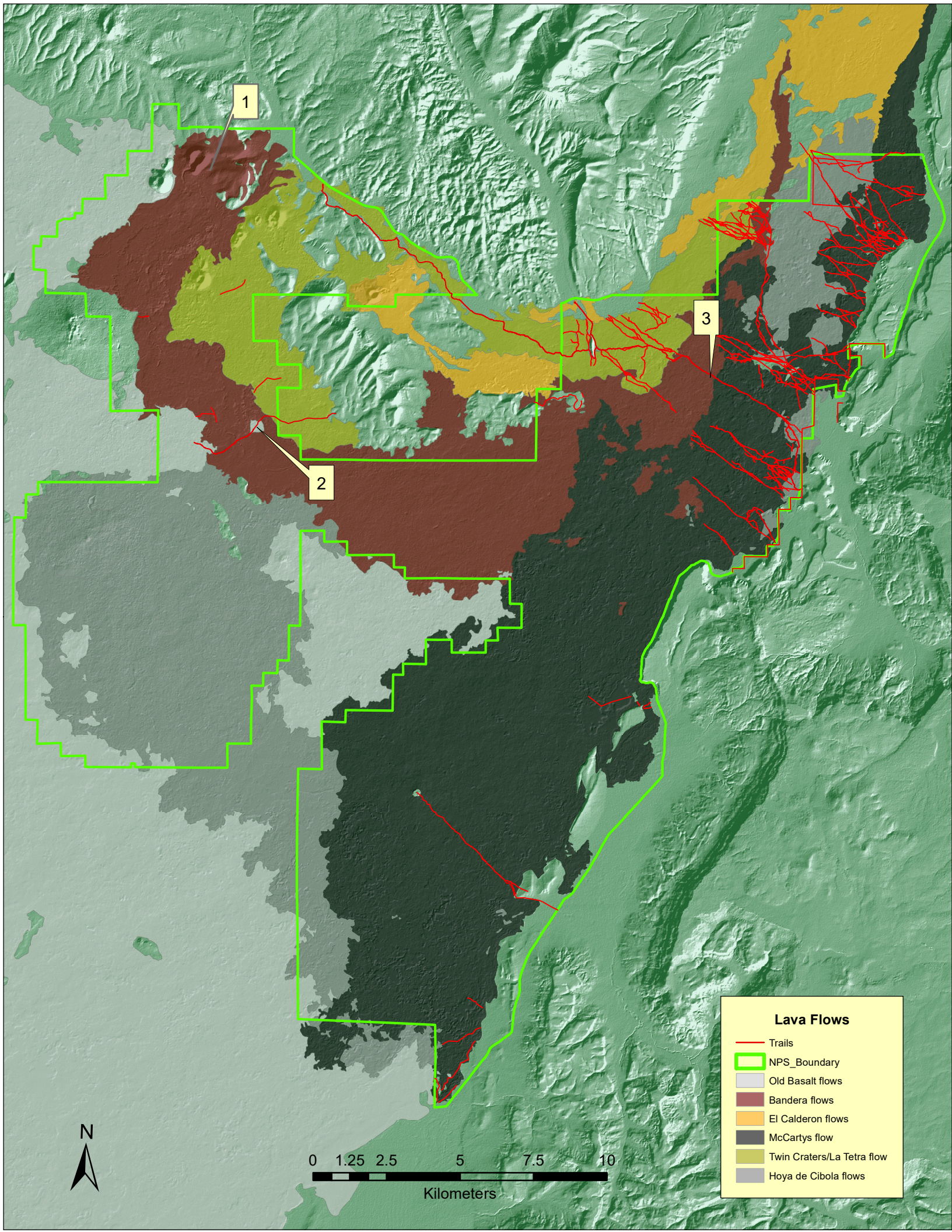

Supplement: Supplementary file 2 — Supplementary Figure S1. [file 41598_2020_76988_MOESM2_ESM.pdf]

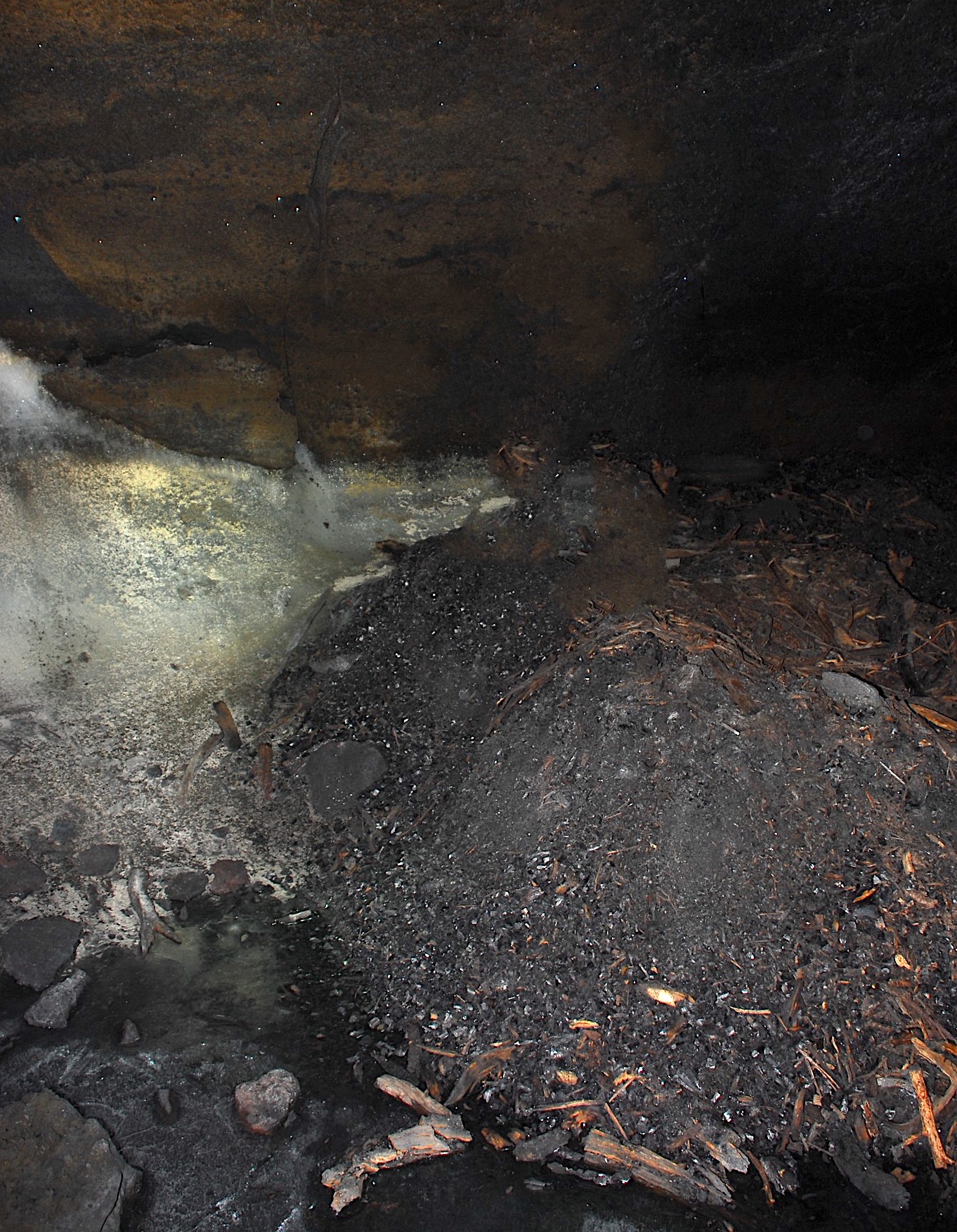

Supplement: Supplementary file 3 — Supplementary Figure S2. [file 41598_2020_76988_MOESM3_ESM.jpg]

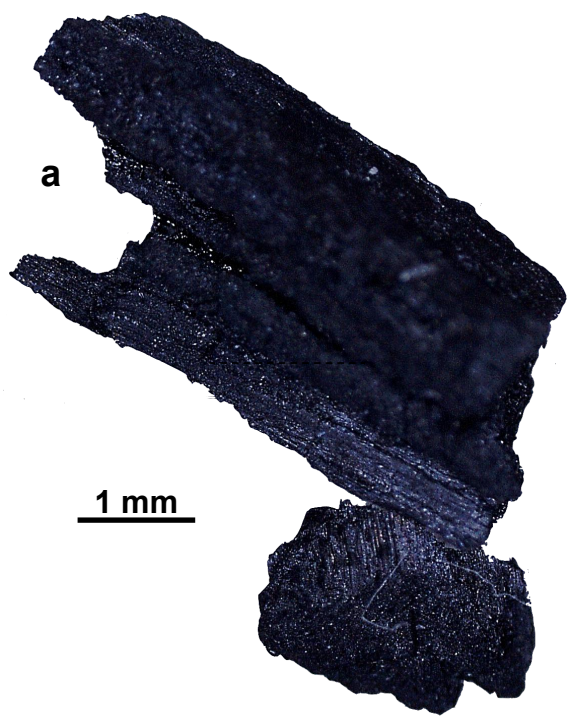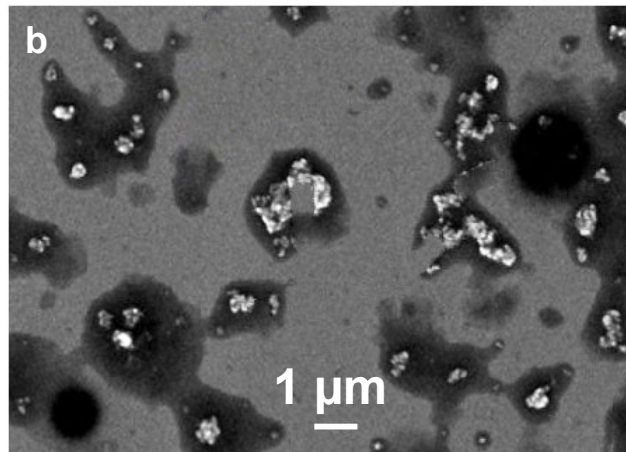

Supplement: Supplementary file 4 — Supplementary Figure S3. [file 41598_2020_76988_MOESM4_ESM.pdf]

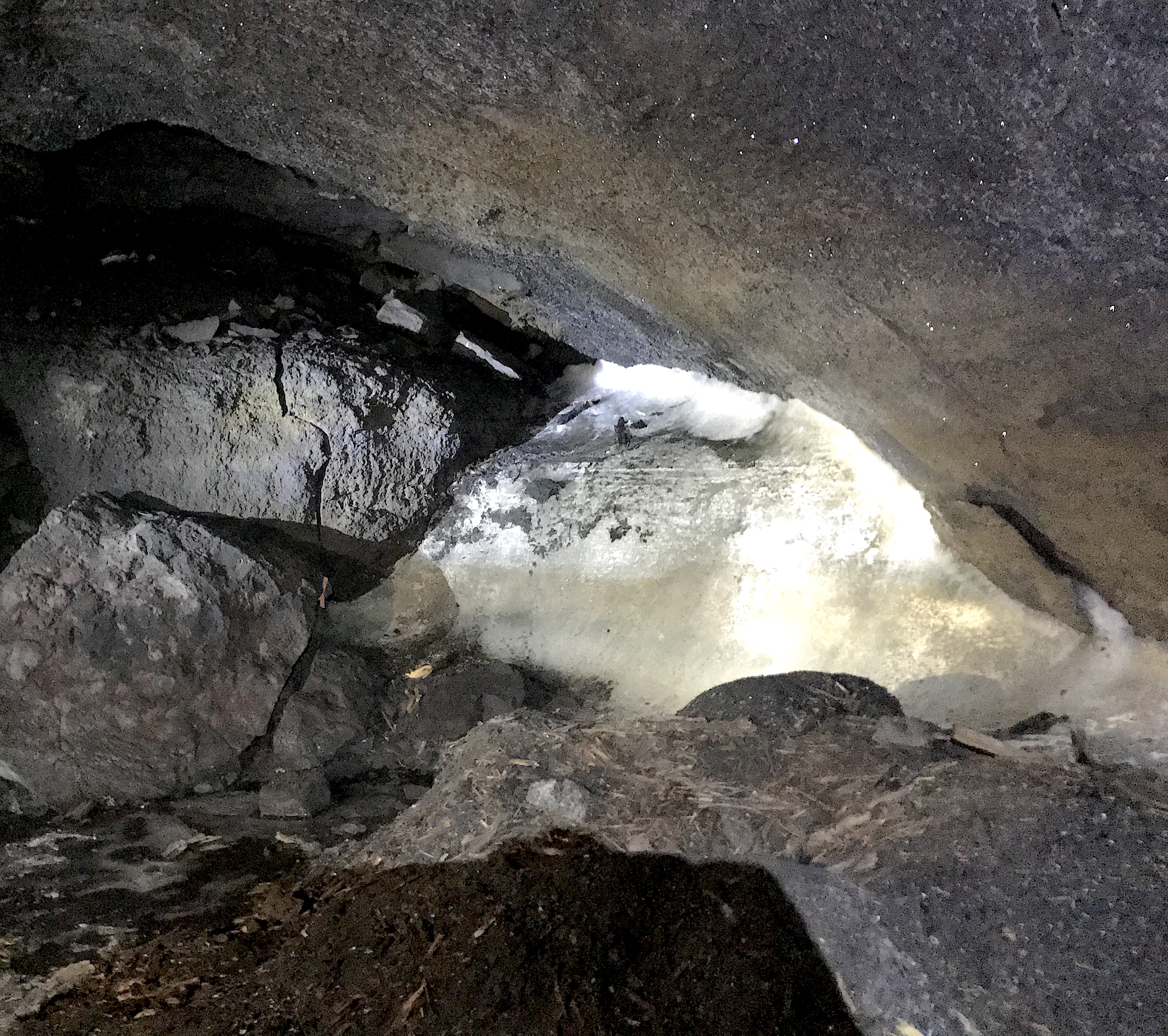

Supplement: Supplementary file 5 — Supplementary Figure S4. [file 41598_2020_76988_MOESM5_ESM.jpeg]

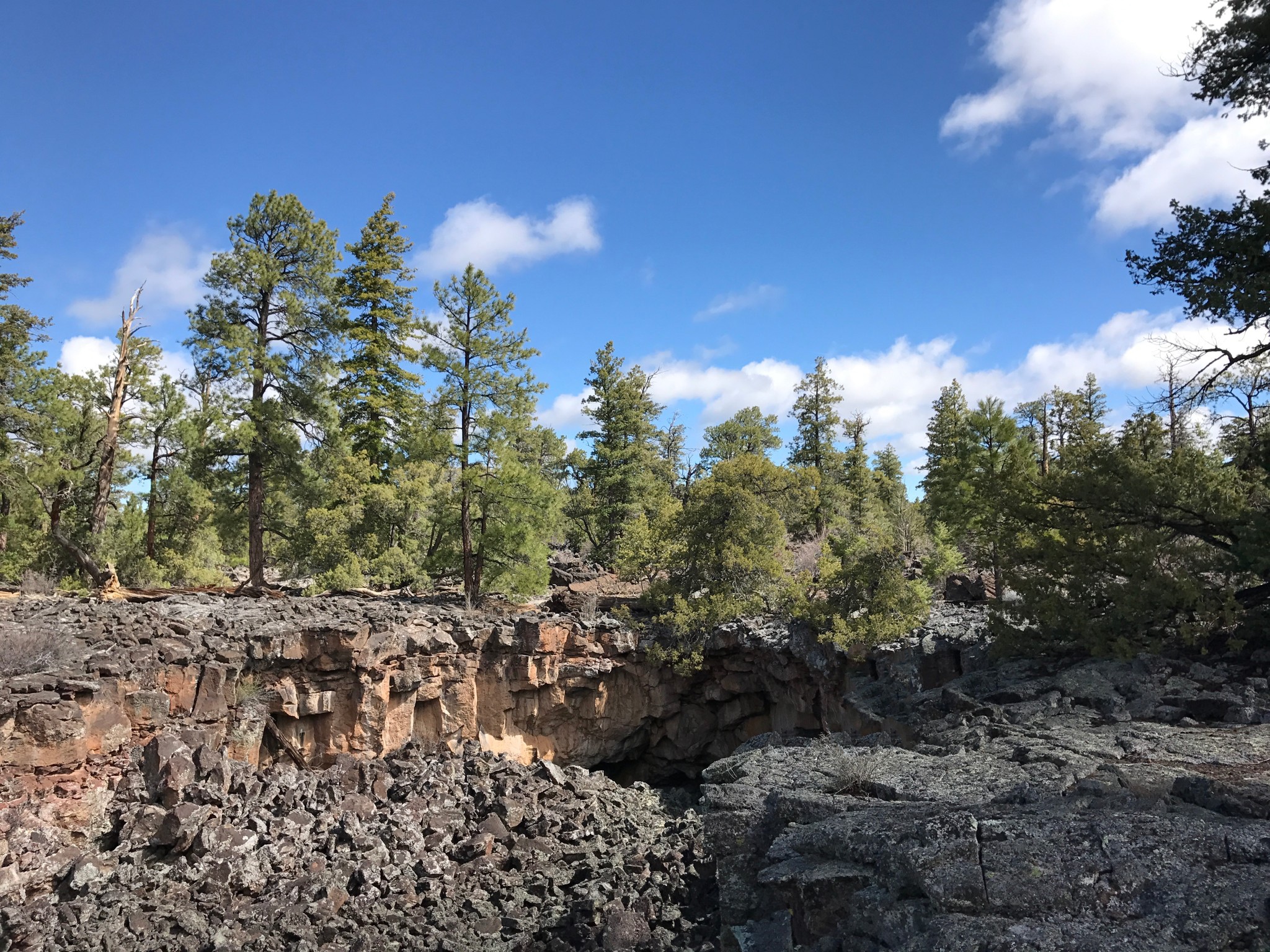

Supplement: Supplementary file 6 — Supplementary Figure S5. [file 41598_2020_76988_MOESM6_ESM.jpg]
