# Supplementary material for: Late Holocene droughts and cave ice harvesting by Ancestral Puebloans
Source: Sci Rep. 2020 Nov 18;10:20131. doi: 10.1038/s41598-020-76988-1 (PMC7674407; doi:10.1038/s41598-020-76988-1)
Supplement: Supplementary file 7 — Supplementary Figure S6. [file 41598_2020_76988_MOESM7_ESM.pdf]

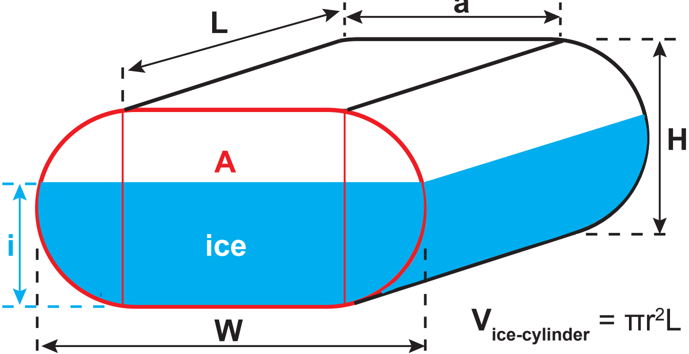

$A = \pi r^2 + 2ra$ $r = H \div 2;\ a = W - H$

$V_{tot} = (\pi r^2 + 2ra) \cdot L$ **(Eq. 1)**

$V_{ice} = V_{ice\text{-}cylinder} + V_{ice\text{-}rectangle}$ **(Eq. 2)**

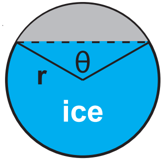

$V_{ice\text{-}cylinder} = \pi r^2 L - (0.5) r^2 (\theta - \sin\theta)$ (Eq. 3)
